# 102. Evaluation of the Association between the Antibiotic Spectrum Index and Antibiotic Days of Therapy: A Retrospective Study across 124 Acute-care Hospitals

**DOI:** 10.1093/ofid/ofab466.304

**Published:** 2021-12-04

**Authors:** Satoshi Kakiuchi, Michihiko Goto, Fernando Casado-Castillo, Eli N Perencevich, Daniel J Livorsi

**Affiliations:** 1 University of Iowa/Iowa City VAMC, Iowa City, Iowa; 2 University of Iowa Carver College of Medicine, Iowa City, Iowa; 3 University of Iowa, Iowa City, Iowa; 4 University of Iowa Carver College of Medicine and Iowa City VA Health Care System, Iowa City, Iowa

## Abstract

**Background:**

Antibiotic stewardship programs often measure antibiotic days of therapy (DOT), but this metric does not reflect the antibiotic spectrum. In this study, we used the previously published Antibiotic Spectrum Index (ASI), which attaches a score (1-13) to the spectrum of each antibiotic, to evaluate the content of antibiotic use across all Veterans Health Administration (VHA) hospitals. We also assessed how benchmarking hospital performance changed when ASI was used instead of DOT.

**Methods:**

We conducted a retrospective cohort study of patients admitted to 124 acute-care VHA hospitals during 2018. We obtained data on administered antibiotics, the days of antibiotic use (DOT), and days-present (DP) from the VHA Corporate Data Warehouse and then aggregated data to the hospital-level using the National Healthcare Safety Network’s methodology. We modified the original ASI by changing 3.8% of the bug-drug scores to ensure consistency across all scores and adding 27 new antibiotics agents. For each hospital, we calculated ASI/DOT, ASI/1,000 DP, and DOT/1,000 DP and ranked hospitals on their performance. We performed a Spearman’s rank-order correlation to compare hospitals on these metrics and their associated rankings.

**Results:**

At the hospital-level, the median ASI/DOT, ASI/1,000 DP and DOT/1,000 DP were 5.4 (interquartile range: 5.2-5.8), 2,332.7 (1,941.8-2,796.2) and 443.5 (362.5-512.2), respectively. There was a strong correlation between the ASI/1,000 DP and DOT/1,000 DP metrics [Spearman’s correlation test: r=0.97 (p< 0.01)] but only a weak and insignificant correlation between ASI/DOT and DOT/1,000 DP [r=0.17 (p=0.06), Figure 1]. Twenty (16.1%) hospitals showed a difference of 10% or more in their ranking for ASI/1,000 DP compared to their ranking for DOT/1,000 DP. The range of ranking difference was from -17.7% to 21.0% (Figure 2a and b).

Figure 1. Distribution of the Antibiotic Spectrum Index / Day of Therapy by Days of Therapy / 1000 Days Present for 124 Acute-Care VHA Hospitals during 2018. Black line: Median values of DOT/1,000 DP and ASI/DOT, respectively.

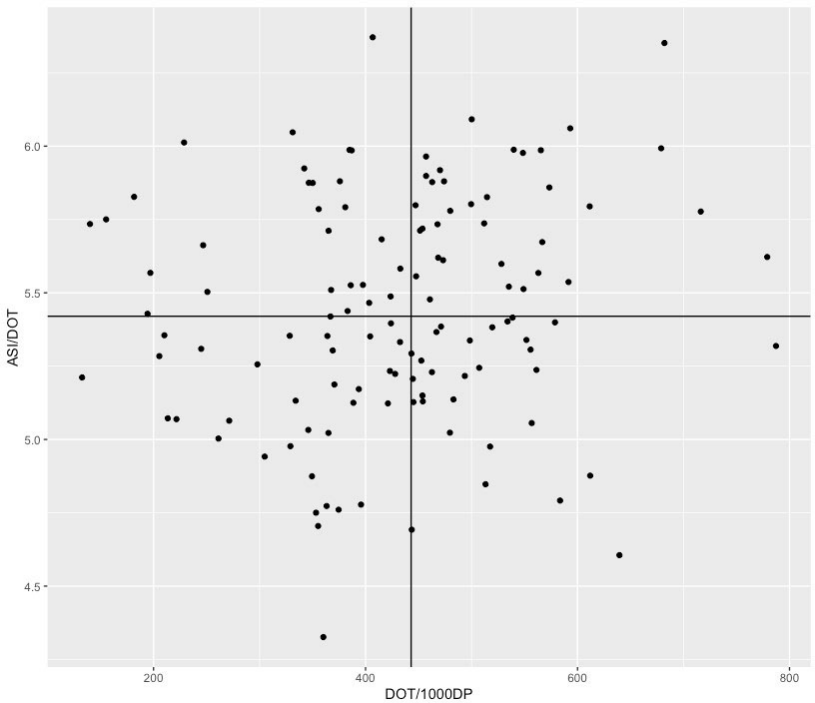

Figure 2. (a) Distribution of the rankings in DOT/1,000 DP and ASI/1,000 DP. Blue line: the position of same ranking between ASI/1,000 DP and DOT/1,000 DP. (b) Distribution of the differences in each hospital’s ranking for DOT/1,000 DP and ASI/1,000 DP

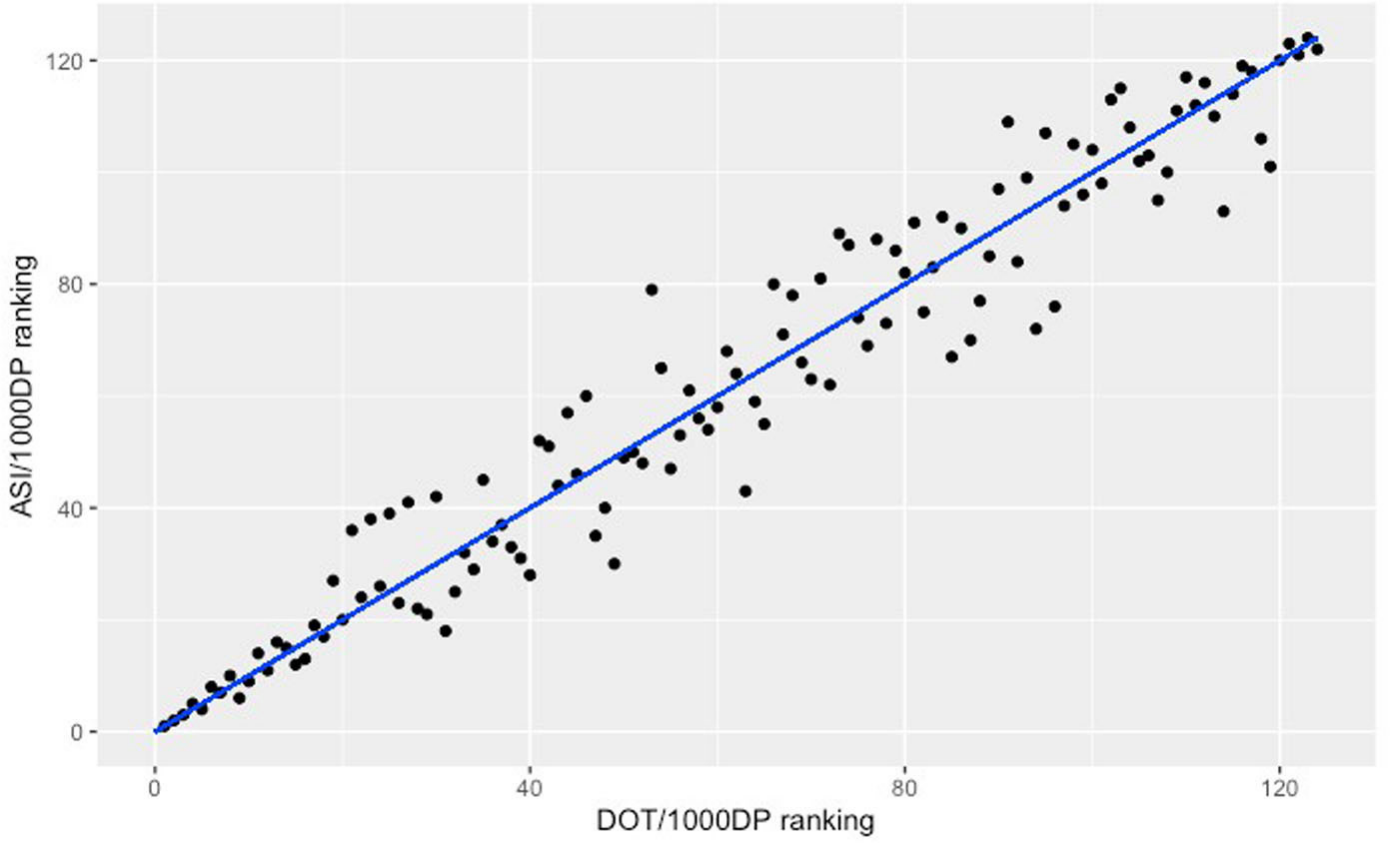

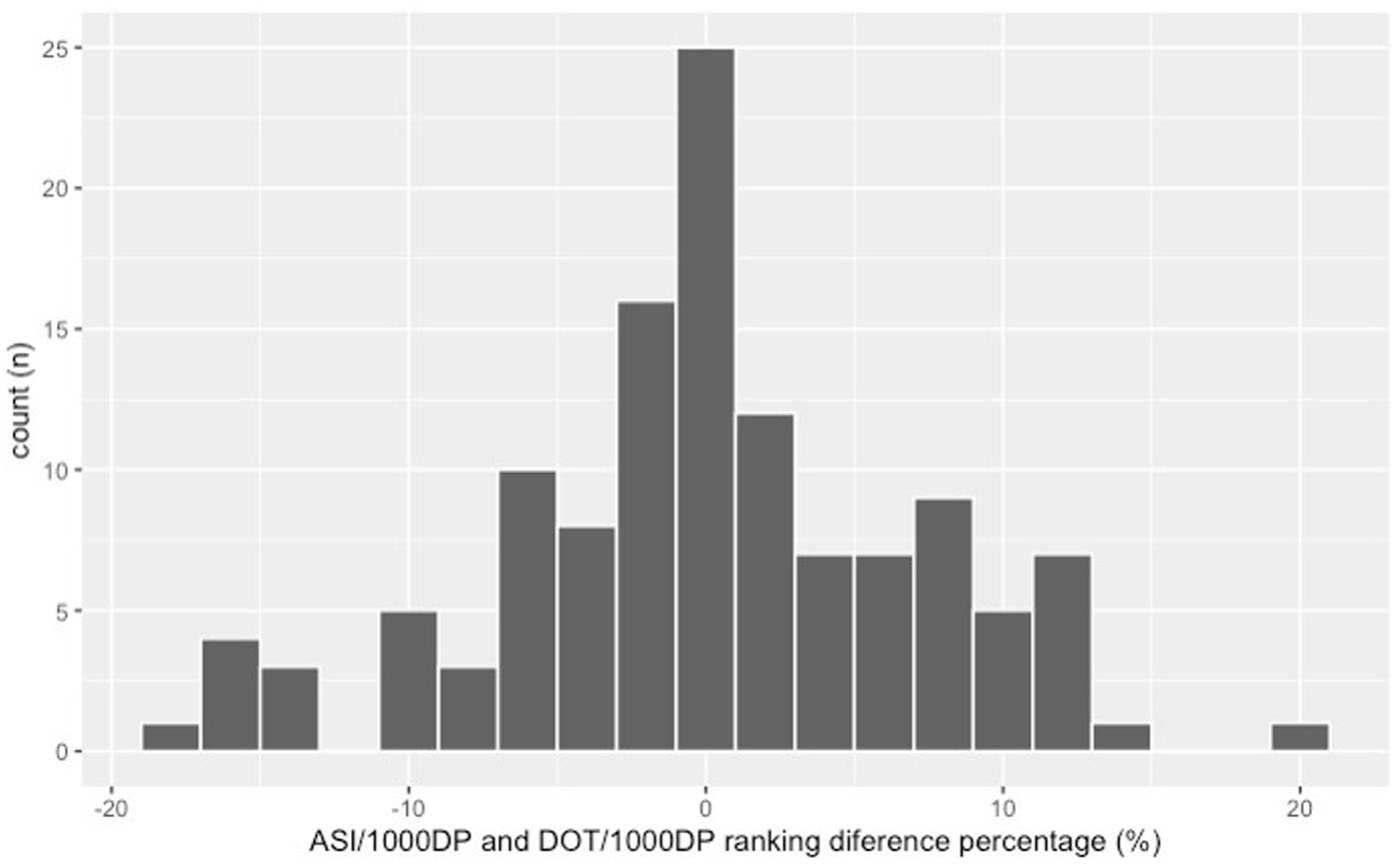

**Conclusion:**

Our findings suggest that hospitals using fewer days of antibiotic therapy did not necessarily use narrower-spectrum antibiotics. ASI/1,000 DP, as a combined measure of antibiotic consumption quantity and average spectrum, provided a different view of hospital performance than DOT/1,000 DP alone. Future work is needed to define how this new metric relates to the quality of antibiotic use.

**Disclosures:**

**All Authors**: No reported disclosures

